# Supplementary material for: Multimodal tactile sensing fused with vision for dexterous robotic housekeeping
Source: Nat Commun. 2024 Aug 11;15:6871. doi: 10.1038/s41467-024-51261-5 (PMC11316753; doi:10.1038/s41467-024-51261-5)
Supplement: Supplementary file 1 — Supplementary Information [file 41467_2024_51261_MOESM1_ESM.pdf]

Supplementary Materials for

Multimodal tactile sensing fused with vision for dexterous robotic housekeeping

The PDF file includes:

Supplementary Text
Figure S1. The constant temperature difference circuit for tactile sensor.
Figure S2. The temperature effect on the tactile sensor.
Figure S3. The effect of contact pressure on the top sensing layer.
Figure S4. Repeatability test of the tactile sensor under 1000 cycles
Figure S5. Microscopic images of different materials.
Figure S6. FFT analyses of the micro features at 1 mm/s for different materials.
Figure S7. Photographs of ten kinds of fabrics.
Figure S8. Tactile responses and micrographs of ten kinds of fabrics.
Figure S9. The calibration method used for the camera.
Figure S10. Target object recognition and object localization for different objects.
Figure S11. The determination of the grasping pose.
Figure S12. The interface signal response when the robot grasps different cups.
Figure S13. Temperature sensing for the amount of the hot water in a cup.
Figure S14. The photographs of ten objects used for a desk-cleaning task in daily life.
Figure S15. The fabrication process of the tactile sensor.
Table S1. Thermal properties of some materials.

Supplementary Text

Temperature measurement and temperature compensation of the tactile sensor

The top sensing layer and the bottom sensing layer have the same structure and measurement principles. For the sake of simplicity, we will use the bottom sensing layer as an example to explain here.

As shown in the Figure S1, due to the high resistance of the cold-film (~ 500 Ω), its heat power can be ignored. Therefore, its temperature is nearly the same as the ambient temperature. And its resistance can be expressed as:

$$R_c = R_{c0}(1 + \alpha_c T) \tag{1}$$

where R_{c0} is the resistance of the cold-film at 0 °C, α_c is its temperature coefficient resistance (TCR), and T represents the ambient temperature. According to the principle of voltage division in circuits, we can obtain:

$$\frac{U_p - U_c}{U_c} = \frac{R_b}{R_t + R_c} \tag{2}$$

U_p, U_c, R_b, R_t refers to the corresponding symbols in Figure S1. Combining equation (1) and equation (2), it can be obtained that the ambient temperature T satisfies:

$$\frac{U_c}{U_p - U_c} = \frac{R_{c0} \cdot \alpha_c}{R_b} \cdot T + \frac{R_t + R_{c0}}{R_b} \tag{3}$$

Here, we define:

$$\begin{cases} \eta = \dfrac{U_c}{U_p - U_c} \\ A = \dfrac{R_{c0} \cdot \alpha_c}{R_b} \\ B = \dfrac{R_t + R_{c0}}{R_b} \end{cases} \tag{4}$$

Then equation (3) can be simplified as:

$$\eta = A \cdot T + B \tag{5}$$

where A and B are constants, so the ambient temperature can be measured using the linear relationship between η and T.

Similarly, since the top sensing layer is in direct contact with the object, it can measure the object temperature.

When the Wheatstone bridge is balanced by the constant temperature difference circuit shown in Figure S1, their resistances satisfiy the following relationship:

$$\frac{R_a}{R_b} = \frac{R_h}{R_t + R_c} \tag{6}$$

R_a refers to the corresponding symbols in Figure S1. It is assumed that the temperature difference between the hot film and the ambient temperature is ΔT. Therefore, we can obtain:

$$R_h = R_{h0}\big(1 + \alpha_h(T + \Delta T)\big) \tag{7}$$

Then the equation (6) can be expressed as:

$$\frac{R_a}{R_b} = \frac{R_{h0}\big(1 + \alpha_h(T + \Delta T)\big)}{R_t + R_{c0}(1 + \alpha_c T)} \tag{8}$$

where R_{h0} and R_{c0} are the resistances of the hot-film and the cold-film at 0 °C respectively, α_h and α_c are the temperature coefficient of resistances (TCRs) of the hot-film and the cold-film respectively. Since the hot-film and cold-film are fabricated using the same material (Pt) and the same fabrication process, α_h and α_c are almost the same, and thus they are collectively recorded as α. In this circuit, we set:

$$\begin{cases} R_a = R_{h0} \\ R_b = R_{c0} \\ R_t = \Delta T \cdot \alpha \cdot R_{c0} \end{cases} \tag{9}$$

which ahieves the balance of the Wheatstone bridge:

$$\frac{R_a}{R_b} = \frac{R_{h0}}{R_{c0}} = \frac{R_{h0}\big(1 + \alpha(T + \Delta T)\big)}{R_{c0}\big(1 + \alpha(T + \Delta T)\big)} = \frac{R_{h0}(1 + \alpha(T + \Delta T))}{R_{c0}(1 + \alpha T) + R_{c0} \cdot \alpha \cdot \Delta T} = \frac{R_h}{R_c + R_t} \tag{10}$$

Due to:

$$\Delta T = \frac{R_t}{\alpha \cdot R_{c0}} \tag{11}$$

R_a, R_b, R_t are the fixed-value resistors mounted on the circuit board out of the sensor device, and they have very low temperature coefficient of resistance (TCR=5 ppm/°C). Therefore, they are barely temperature-sensitive. This shows that the temperature difference ΔT between the hot-film and the environment is constant and not affected by the ambient temperature. In other words, the temperature compensation is realized by this balanced Wheatstone bridge circuit.

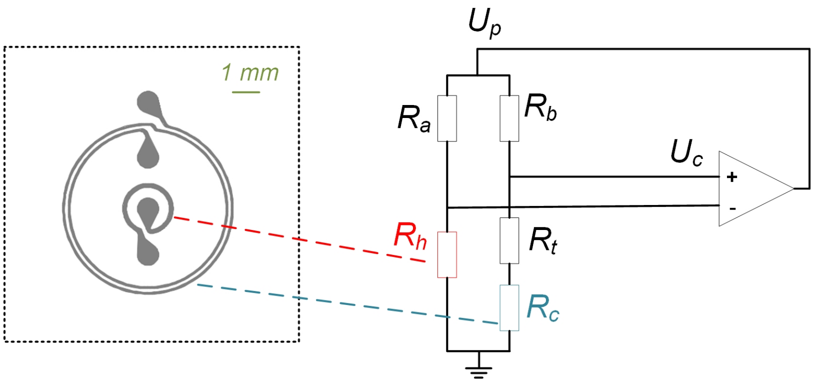

Figure S1. The constant temperature difference circuit for tactile sensor. Both the top sensing layer and the bottom sensing layer use the circuit shown in the figure, which has a temperature compensation function. The closed-loop feedback control circuit ensures self-sustaining constant temperature difference between the hot-film R_h and cold-film R_h. Therefore, the tactile sensor achieves accurate measurements of multiple information immune from environment temperature.

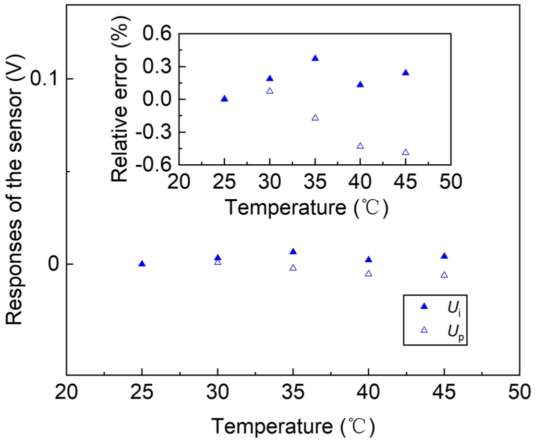

Figure S2. The temperature effect on the tactile sensor. When the ambient temperature changes from 25 °C to 45 °C, the output voltage of the tactile sensor remains stable (less than 0.6%), allowing it resistant to environmental temperature variation.

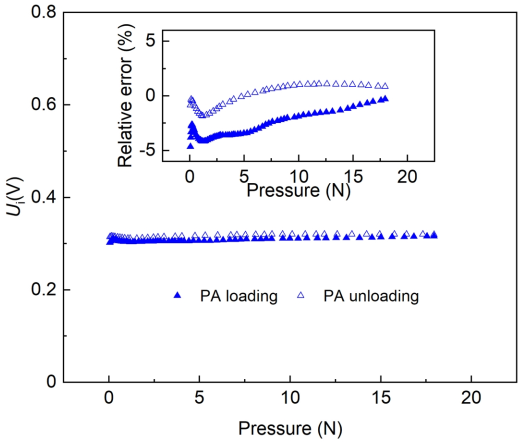

Figure S3. The effect of contact pressure on the top sensing layer. The crosstalk is less than 4.7%, which shows that the contact pressure basically does not affect the slip detection of the top sensing layer.

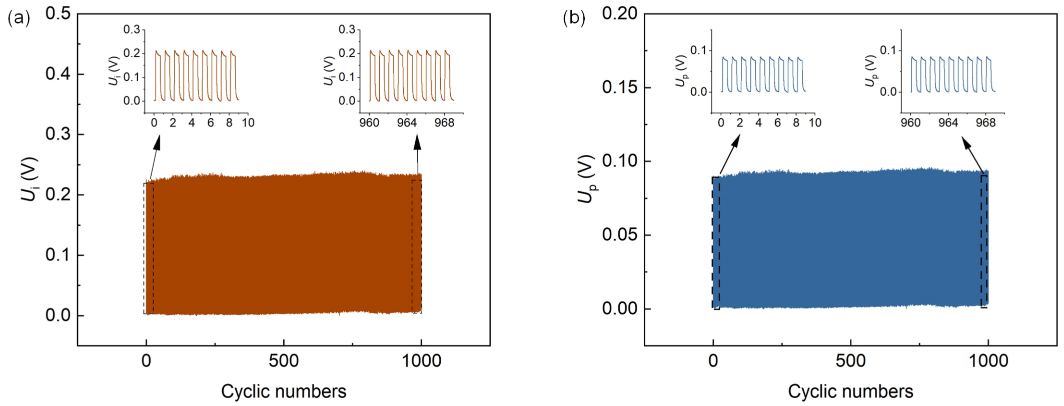

Figure S4. Repeatability test of the tactile sensor under 1000 cycles. (a) Response of the top sensing layer. (b) Response of the bottom sensing layer. Under 1000 cycle tests, the output of the sensor remained basically unchanged, proving the high reliability of the sensor.

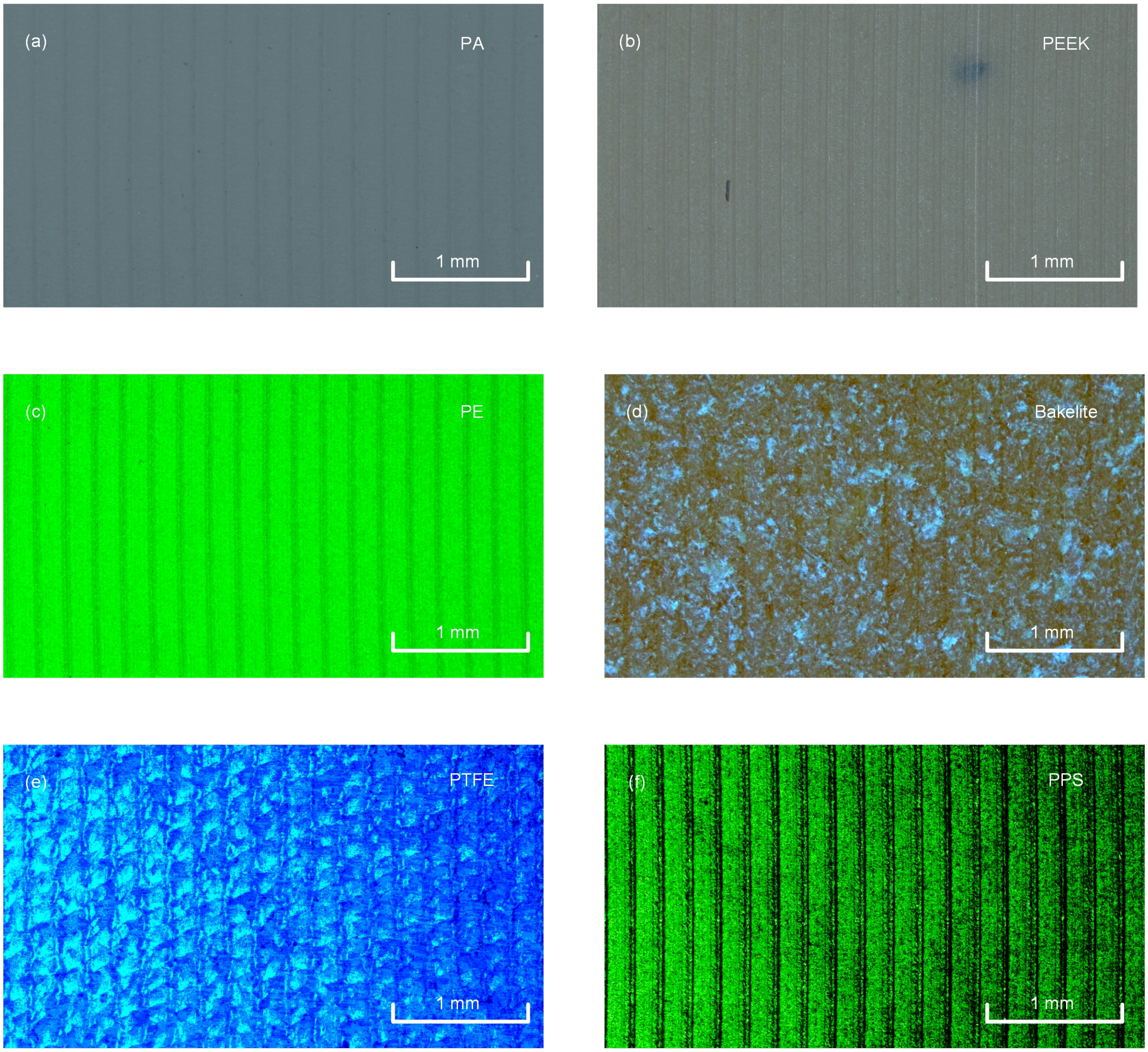

Figure S5. Microscopic images of different materials. (a) PA. (b) PEEK. (c) PE. (d) Bakelite. (e) PTFE. (f) PPS. It can be seen from the figure that the grating periods on the surface of these materials are basically consistent with that measured by the tactile sensor.

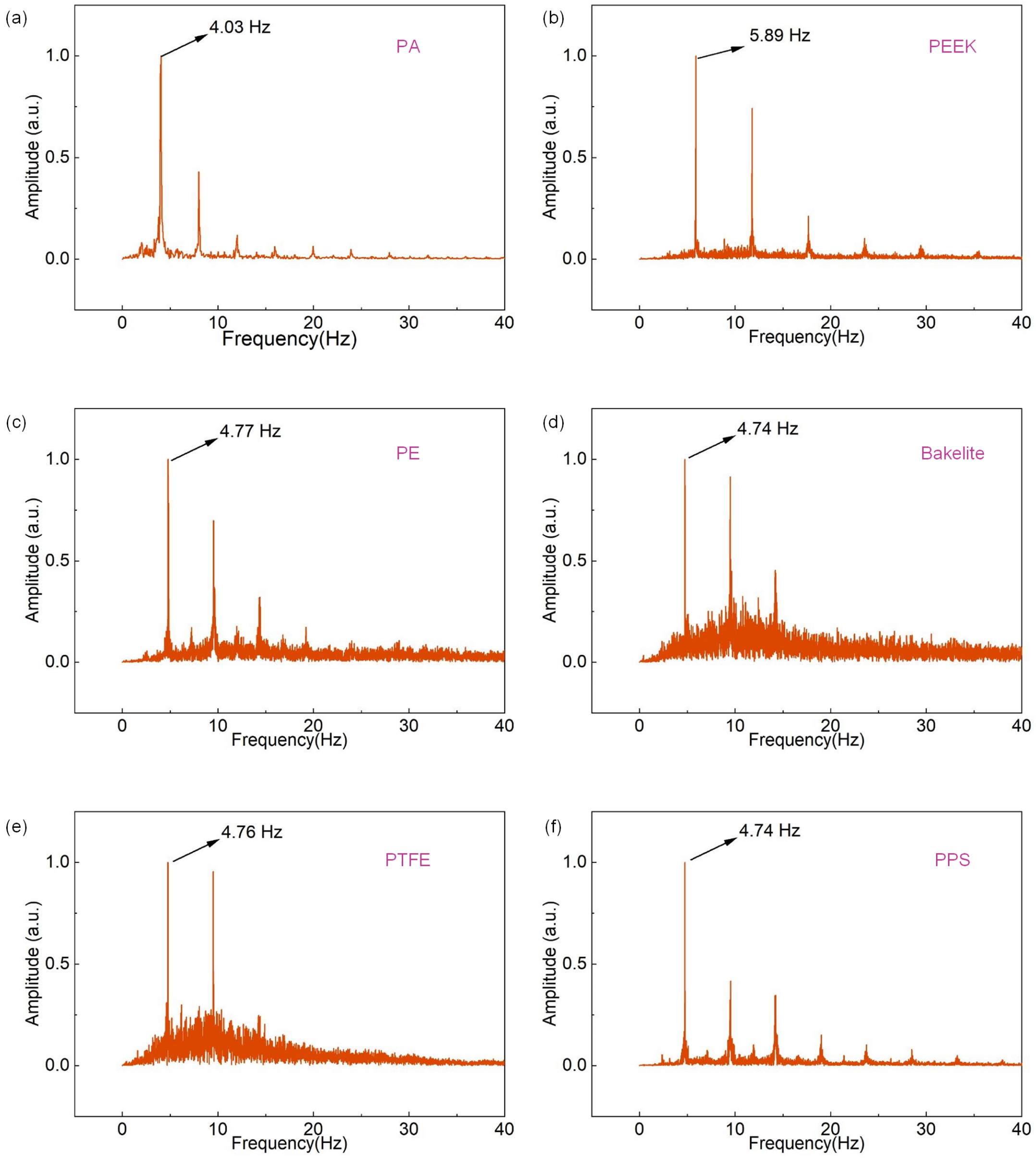

Figure S6. FFT analyses of the micro features at 1 mm/s for different materials. (a) The fundamental frequency of PA is 4.03 Hz. (b) The fundamental frequency of PEEK is 5.89 Hz. (c) The fundamental frequency of PE is 4.77 Hz. (d) The fundamental frequency of Bakelite is 4.74 Hz. (e) The fundamental frequency of PTFE is 4.76 Hz. (f) The fundamental frequency of PPS is 4.74 Hz. Performing a fast Fourier transform (FFT) analysis on these signals in the frequency domain yields the spectrum shown in this figure. From this figure, we deduce that the grating period on the surface of PA, PEEK, PE, Bakelite, PTFE, and PPS are approximately 248.1, 170.8, 209.6, 211.0, 210.1, and 211.0 μm, respectively.

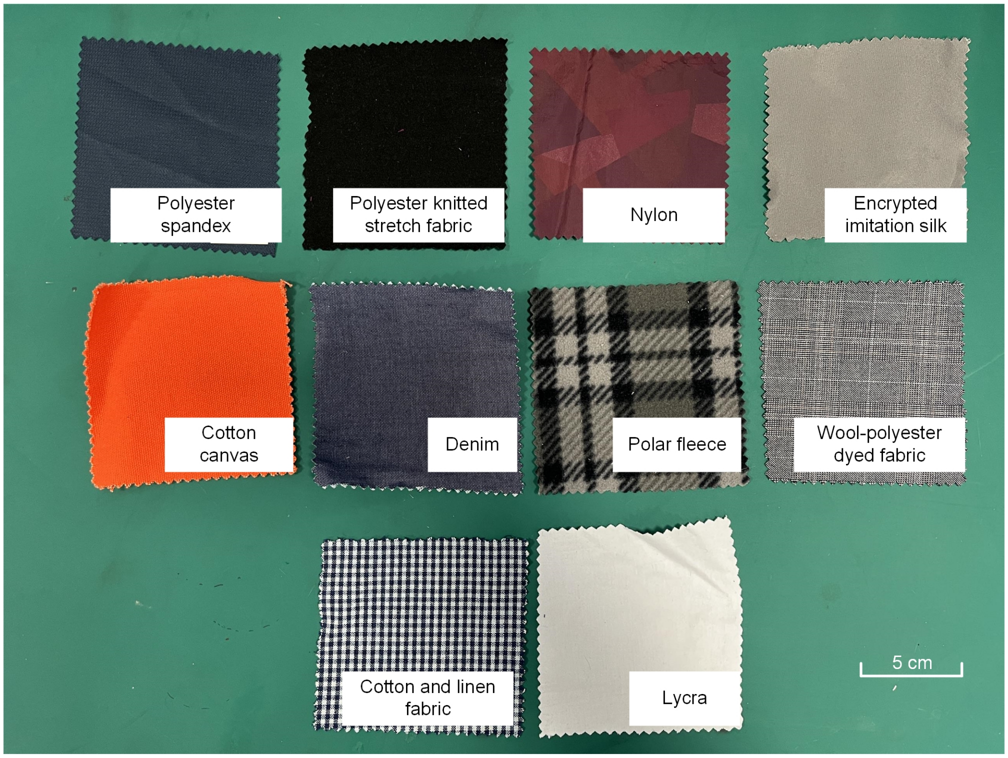

Figure S7. Photographs of ten kinds of fabric. These fabric (polyester spandex, polyester knitted stretch fabric, nylon, encrypted imitation silk, cotton canvas, denim, polar fleece, wool-polyester fabric, carton and linen fabric, and lycra) are used for fabric recognition, with an total accuracy of 94.3%.

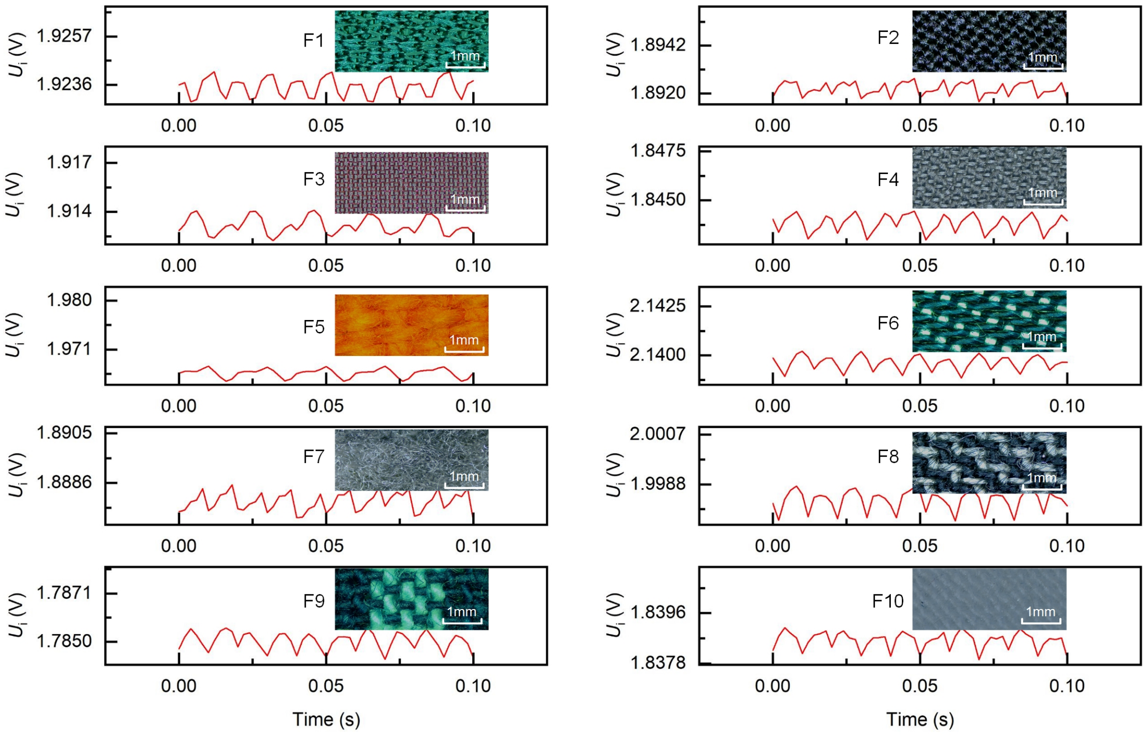

Figure S8. Tactile responses and micrographs of ten kinds of fabric.

(a)

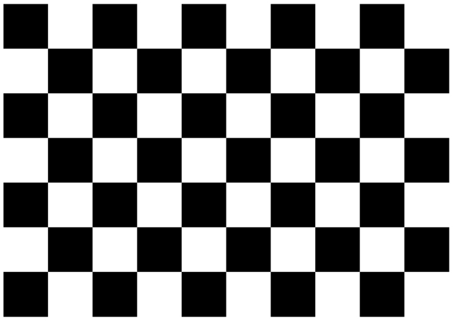

(b)

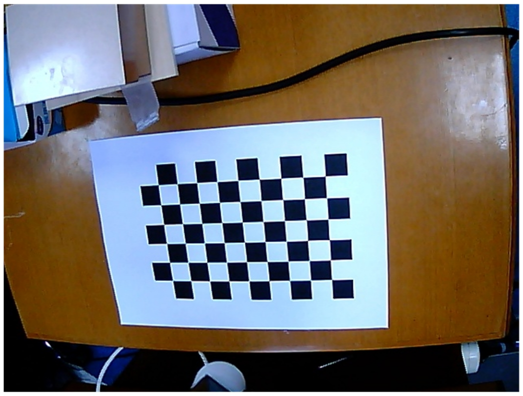

Figure S9. The calibration method used for the camera. (a) The calibration chessboard with 7 × 10 squares (each square is 20 mm × 20 mm). (b)The photograph taken in one typical orientation.

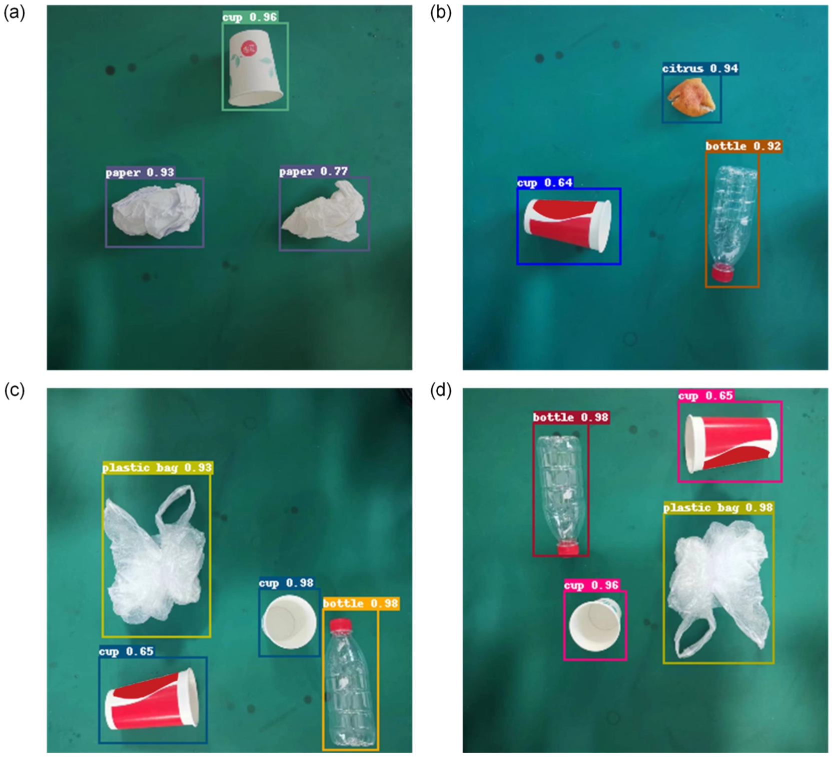

Figure S10. Target object recognition and object localization for different objects. (a) The recognized objects are a cup, a paper, and another paper with the confidence probabilities of 0.96, 0.93, and 0.77, respectively. (b) The recognized objects are a citrus, a cup, and a bottle with the confidence probabilities of 0.94, 0.64, and 0.92, respectively. (c) The recognized objects are a plastic bag, a cup, another cup, and a bottle with the confidence probabilities of 0.93, 0.98, 0.65, and 0.98, respectively. (d) The recognized objects are a bottle, a cup, another cup, and a plastic bag with the confidence probabilities of 0.98, 0.65, 0.96, and 0.98, respectively.
This figure shows the scene where the robot initially recognizes and locates a variety of different objects on the table by vision. The panels in the figure display the results of object localization and recognition, as well as the corresponding confidence probabilities. For example, "cup 0.96" indicates that the recognized object is a paper cup and its confidence probability is 0.96.

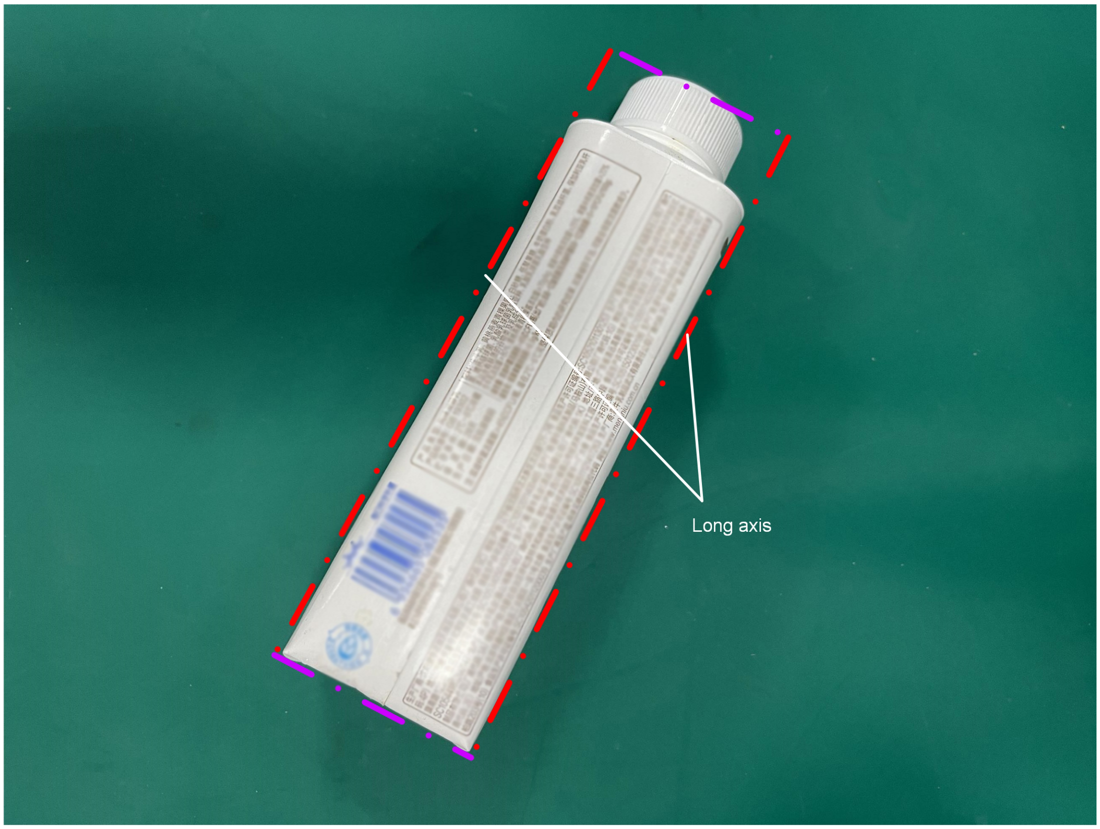

Figure S11. The determination of the grasping pose. First, object detection is used to determine every object's position and its rectangle box as mentioned before. The stereo camera can acquire object's height to the desk which can be used to recognize the cup's placement state. The object's outline also can be acquired using edge detection based on depth. Then, linear fitting is adopted for the outline points to obtain the estimation of the object's long axis. The robot grasping pose can be determined based on the direction of the long axis.

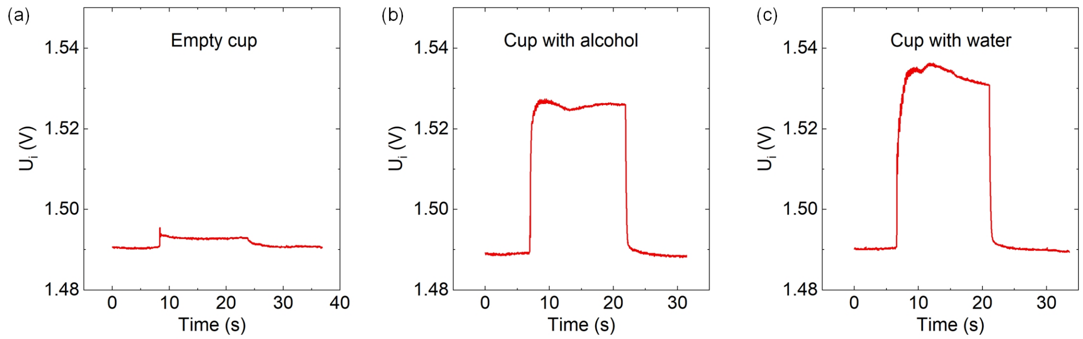

Figure S12. The interface signal response when the robot grasps different cups. (a) Empty cup. (b) A cup with alcohol. (c) A cup with water. Due to the different valuse of thermal conductivity (water > alcohol > paper cup) (shown in Table S1), the heat transfer between the tactile sensor and the object is also different. Therefore, the interface sensing signal of the sensor also has the same relationship: water > alcohol > paper cup.

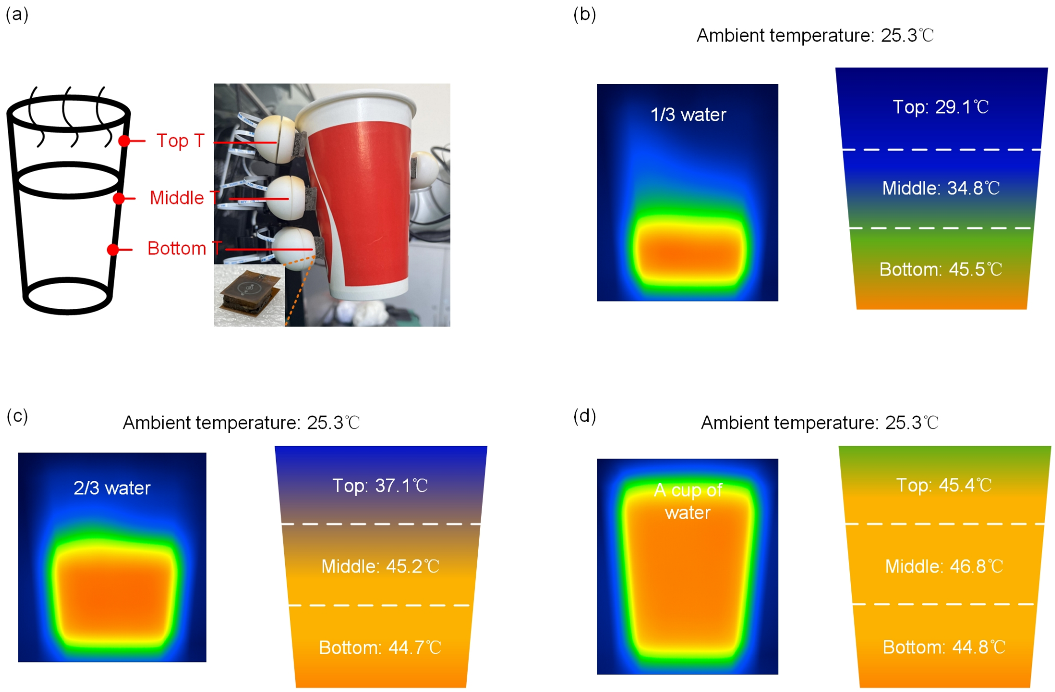

Figure S13. Temperature sensing for the amount of the hot water in a cup. (a) A robotic hand integrated with three tactile sensors is used to perceive the amount of water in the cup, where three sensors measure the temperature of the top, middle, and bottom of the cup. The small picture shows the photograph of the tactile sensor. (b) The temperature distribution map for 1/3 hot water measured from the infrared camera and the tactile sensor. (c) The temperature distribution map for 2/3 hot water measured from the infrared camera and the tactile sensor. (d) The temperature distribution map for a cup of hot water measured from the infrared camera and the tactile sensor.

As shown in Figure S8a, we integrate a robotic hand with three tactile sensors to sense the amount of hot water in a cup. During the grasping process, the tactile sensors can measure the temperature of three contact points: top, middle, bottom. As shown in Figure S8b, we use a thermal infrared (IR) camera to measure the temperature distribution of one-third cup of hot water on the left. The image on the right demonstrates that we divide the temperature gradient equally between adjacent measuring points to obtain an approximate temperature distribution map, which is similar to the result obtained by the infrared camera. The results indicate that the bottom tactile sensor detects a temperature (45.5°C) significantly higher than room temperature, while the middle and top tactile sensors measured a temperature slightly higher than room temperature (34.8°C and 29.1°C respectively). As the amount of hot water in the cup increases to two-thirds and a full cup, the high temperature area in the temperature distribution map measured from the infrared camera and the tactile sensor increases simultaneously and remains consistent (Figure S8c and S8d). This demonstrates that the manipulator equipped with the tactile sensor can reliably perceive the amount of hot water in the cup.

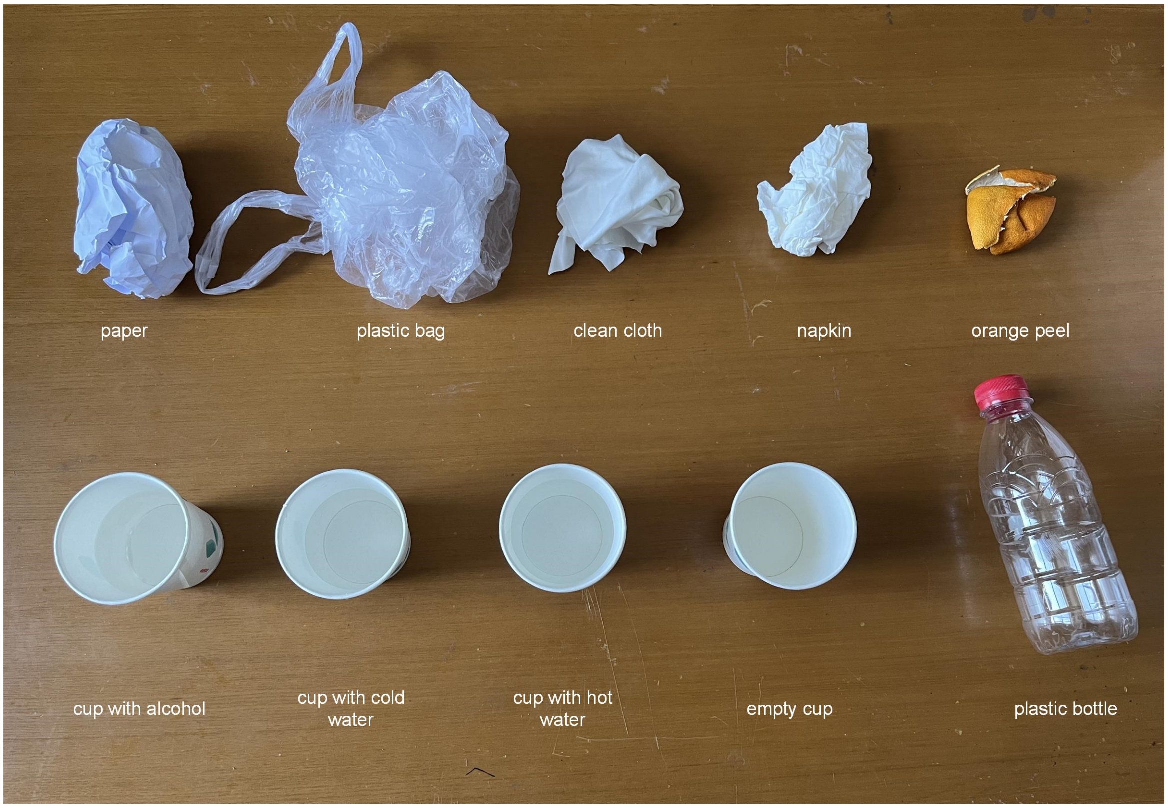

Figure S14. The photographs of ten objects used for a desk-cleaning task in daily life. They are paper, cleaning cloth, napkin, plastic bag, plastic bottle, orange peel, cup with cold water, cup with alcohol, cup with hot water and empty cup.

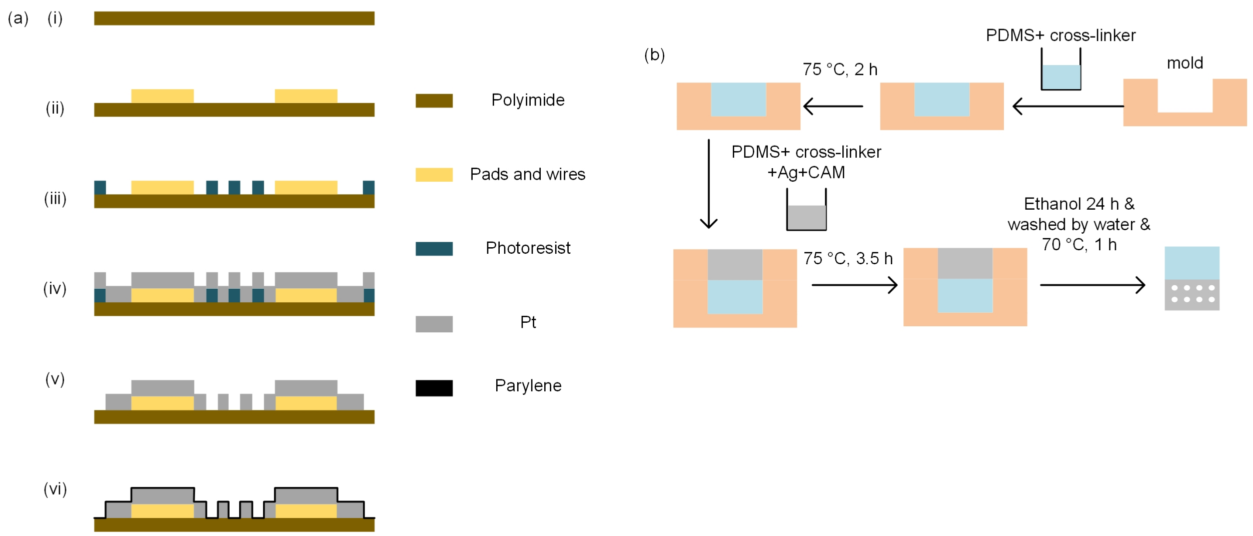

Figure S15. The fabrication process of the tactile sensor. (a) Fabrication process diagram of the top/bottom sensing layer. (b) Fabrication process diagram of the PDMS layer and porous material.

Table S1. Thermal properties of some materials

Material	Thermal conductivity (W/(m·K))
Paper	0.09
Alcohol	0.171
PU	0.180
PPS	0.210
PEEK	0.250
PA	0.290
PE	0.331
Bakelite	0.377
Water	0.598
Steel	16.3
Brass	109
