# Supplementary material for: Multimodal tactile sensing fused with vision for dexterous robotic housekeeping
Source: Nat Commun. 2024 Aug 11;15:6871. doi: 10.1038/s41467-024-51261-5 (PMC11316753; doi:10.1038/s41467-024-51261-5)
Supplement: Supplementary file 3 — Description Of Additional Supplementary File [file 41467_2024_51261_MOESM3_ESM.pdf]

Description of Additional supplementary file

Movie S1. Summary of multimodal tactile sensing fused with vision for dexterous robotic housekeeping.

Movie S2. Tactile feedback enables a stable grasp.

Movie S3. The comparison between tactile-visual fusion method and only vision method.

Movie S4. Case 1 The tactile-visual fusion robot accomplishes a desk-cleaning task.

Movie S5. Case 2 The tactile-visual fusion robot accomplishes another desk-cleaning task.
